# Tubulointerstitial Nephritis and Uveitis Syndrome in a Twelve-Year-Old Girl

**DOI:** 10.1155/2013/652043

**Published:** 2013-04-18

**Authors:** Alessia Paladini, Vittorio Venturoli, Giovanni Mosconi, Loretta Zambianchi, Luigi Serra, Enrico Valletta

**Affiliations:** ^1^Department of Pediatrics, G.B. Morgagni-L. Pierantoni Hospital, AUSL Forlì, Via Carlo Forlanini 34, 47100 Forlì, Italy; ^2^Pediatric Clinic, University of Ferrara, 44121 Ferrara, Italy; ^3^Department of Nephrology, G.B. Morgagni-L. Pierantoni Hospital, AUSL Forlì, Forlì, Italy; ^4^Department of Anatomical Pathology, G.B. Morgagni-L. Pierantoni Hospital, AUSL Forlì, Forlì, Italy

## Abstract

Tubulointerstitial nephritis and uveitis (TINU) syndrome is a rare disorder defined by the combination of biochemical abnormalities, tubulointerstitial nephritis, and uveitis. We describe a 12-year-old female, presented with a ten-day history of fever, characterized by sudden onset and rapid spontaneous resolution in few hours, accompanied by shivering, extreme fatigue, and loss of appetite. Laboratory values were consistent with renal failure of tubular origin. Renal biopsy confirmed a tubulointerstitial nephritis, with acute tubulitis, polymorphonuclear infiltration, and microabscesses. The renal interstitium was occupied by a dense inflammatory infiltrate, consisting of lymphocytes, plasma cells, and neutrophils. Glomerular structures were preserved. Ophthalmological examination that suggested a previous asymptomatic bilateral uveitis and HLA typing (HLA-DQA1∗0101/0201 and HLA-DQB1∗0303/0503) further supported the suspect of TINU syndrome. TINU syndrome is probably an underdiagnosed disorder, responsible for many cases of idiopathic anterior uveitis in young patients, especially in those who have asymptomatic renal disease and when proper diagnostic tests are not performed at the time of presentation.

## 1. Introduction

 Tubulointerstitial nephritis and uveitis (TINU) syndrome is a rare disorder, first described in 1975 by Dobrin et al. [1] in two adolescent female patients with acute eosinophilic interstitial nephritis and anterior uveitis. In 2001, Mandeville et al. [2] reviewed 133 published cases of TINU syndrome showing a median age at presentation of 15 years. Since then, few small descriptive series and some case reports in the ophthalmology and nephrology literature tried to better elucidate the clinical and histopathological features of this inflammatory disease [3–6]. TINU syndrome is defined by the combination of biochemical abnormalities, tubulointerstitial nephritis, and uveitis. Most patients with TINU are adolescents and young women, although it has been reported also in adults and the elderly [7]. There is a female to male predominance (3 : 1) with no particular ethnic affinity [2]. In some instances, prior infection (in particular Chlamydia and Epstein-Barr virus) or the use of specific drugs (antibiotics to treat upper respiratory infections and NSAIDs) has been implicated. TINU syndrome has also been reported in patients with autoimmune diseases like hypoparathyroidism, hyperthyroidism, IgG4-related autoimmune disease, and rheumatoid arthritis [8–11]. The underlying mechanisms for TINU are not well understood. Limited data suggest that modified C-reactive protein (mCRP), an autoantigen common to both the uvea and renal tubular cells, may be involved in the pathogenesis [12]. A strong association with HLA-DQA1∗01, HLA-DQB1∗05, and HLA-DQB1∗01 has been observed [13].

 In this paper, we describe a 12-year-old female who had acute renal insufficiency due to biopsy-proven tubulointerstitial nephritis and bilateral asymptomatic uveitis. Clinical, bioptic, and HLA findings strongly suggested a TINU syndrome.

## 2. Case Report

**Figure 1:**
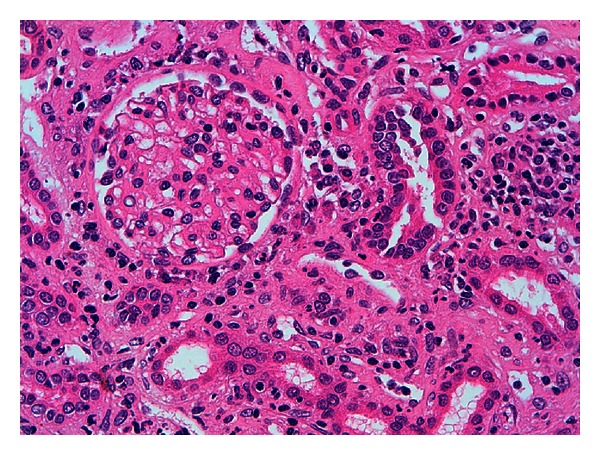
Renal cortex with a complete glomerulus. Mixed inflammatory infiltrate between the cortical tubules (lymphocytes, plasmacells, histiocytes, and polymorphs). Hematoxylin eosin 400x.

**Figure 2:**
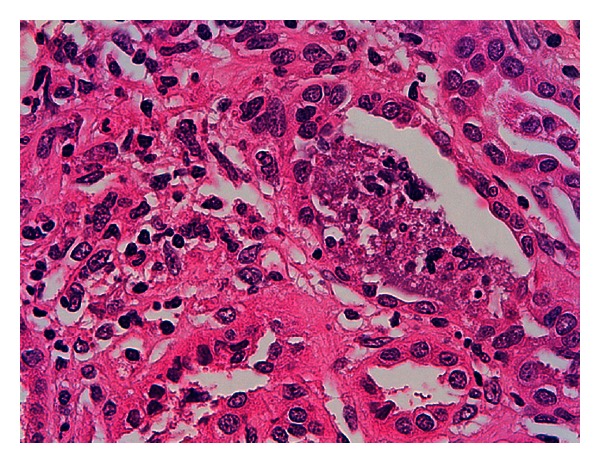
Acute phlogosis inside the tubule. Necrotic debris in the lumen of a tubule. Hematoxylin eosin 630x.

**Figure 3:**
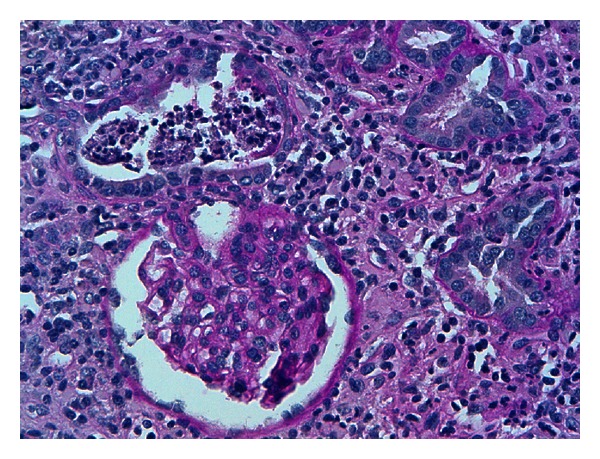
PAS reaction shows a tubular microabscess at the top of the picture and a normal glomerulus at the bottom. PAS 400x.

 A 12-year-old female presented with a ten-day history of fever characterized by sudden onset and rapid spontaneous resolution in a few hours, accompanied by shivering, extreme fatigue, and loss of appetite. She was treated with cefpodoxime (10 mg/kg/day) for a week, with defervescence but worsening of fatigue and weight loss. Her past medical history, as well as family history, was unremarkable. Previous blood and urine tests were normal. She had menarche at 10 years of age with regular menses and only mild dysmenorrhea. On admission, the physical examination was normal, and she was afebrile and normotensive. Laboratory values revealed increased serum creatinine (2.3 mg/dL), metabolic acidosis, nonhemolytic normochromic normocytic anemia (Hb 9.5 g/dL), and low erythropoietin levels (1.6 UI/L, normal values 11–29). ESR (98 mm/h) and CRP (42 mg/dL) were increased. Urinalysis showed urinary pH 6.5, low urine osmolality (394 mOsm/Kg), normoglycemic glycosuria (>500 mg/dL), nonnephrotic proteinuria (0.59 g/L), and microalbuminuria (0.09 g/L) with normal proteinemia, moderate hematuria (38 cells/*μ*L), high values of alpha-1 (0.09 g/Lm n.v. <0.01), and beta-2 microglobulin (3.8 mg/L, n.v. 0.8–2.2). C3 and C4 levels, antinuclear (ANA), and antineutrophil cytoplasmic (ANCA) antibodies were normal. Renal sonography and voiding cystography were unremarkable. Renal biopsy was consistent with tubulointerstitial nephritis, with acute tubulitis and polymorphonuclear infiltration that surrounded the epithelial cells and invaded the lumen forming microabscesses. The renal interstitium was occupied by a dense inflammatory infiltrate, consisting of lymphocytes, plasma cells, and neutrophils. Interstitial fibrosis, eosinophils, granulomas, and signs of vasculitis were virtually absent. Glomerular structures were preserved. At immunofluorescence, specific staining for immunoglobulin A and M at the tubular level was negative (Figures 1, 2, and 3).

 To complete the diagnostic workup of tubulointerstitial nephritis, the patient, though asymptomatic, underwent an ophthalmic examination that identified fine keratic precipitations consistent with previous anterior bilateral uveitis. Visual acuity was unimpaired and posterior segment examination was normal. HLA typing—presence of HLA-DQA1∗0101/0201 and HLA-DQB1∗0303/0503—further supported the suspect of TINU syndrome. Oral prednisone (1 mg/kg) was introduced and continued for 45 days on tapering doses. Serum creatinine normalized within 3.5 months and 5 months later renal function and urinalysis remained normal. Nephrologic and ophthalmologic followup were scheduled.

## 3. Discussion

 Our patient's clinical and biochemical features clearly oriented towards renal failure of tubular origin (metabolic acidosis, increased alpha-1 and beta-2 microglobulin levels, microalbuminuria, glycosuria and low urine osmolality), without any sign of significant glomerular involvement (normal serum immunoglobulins and complement and nonnephrotic proteinuria). Renal biopsy confirmed the acute tubulointerstitial nephritis (AIN) with inflammatory tubulointerstitial involvement that spared glomerular structures. 

 AIN is an important cause of acute renal failure, induced most often by drugs, infective agents, autoimmune disorders, or other systemic diseases and TINU syndrome [14–18] (Table 1). Drug-induced AIN accounts for the majority of interstitial nephropathies, and it should be suspected when the onset of characteristic laboratory findings is temporally related to the exposure to the drug. Patients may have signs of an allergic-type reaction, in particular if the offending agent belongs to the class of beta-lactam antibiotics [14]. The average time between the starting of the treatment and the appearance of renal disease is ten days, ranging from one day (after some antimicrobials) to several months (after NSAIDs) [18]. The pathogenetic mechanism is believed to be immunologic since extrarenal manifestations of hypersensitivity are common, and they are dose unrelated and recur after reexposure to the drug. In most patients, renal disease improves spontaneously in a few weeks after withholding of the responsible drug. In subjects with AIN secondary to infectious disease, extra renal manifestations are dominated by the underlying infection, and a complete serologic and microbiologic diagnostic workup should be done to exclude any acute or chronic cause of infection. Renal biopsy is the definitive method of establishing the diagnosis of AIN [14, 17]. There are no randomized trials to support the use of corticosteroids in the treatment of AIN, but some clinical reports demonstrated rapid improvement of renal function within few days after initiation of therapy. Early steroid administration (usually prednisone 1 mg/kg/day for two or three weeks, followed by gradually tapering dose over three or four weeks) is likely to reduce the number and extent of inflammatory infiltrates, thus decreasing the risk of subsequent fibrosis [19].

 In our patient, systemic symptoms (fever, fatigue, and weight loss) preceded the cefpodoxime treatment, and this excluded the possibility of a drug-induced AIN. Also the hypothesis of an AIN secondary to infectious disease was unlikely, since there were no extra renal symptoms suggestive of an ongoing infectious process and the serological survey for common causative agents of AIN was negative. The age and the sex of our patient, the detection of anterior uveitis and the presence of predisposing HLA were all consistent with the diagnosis of TINU syndrome. Remarkably, she was completely asymptomatic from an ophthalmic point of view and ocular findings only witnessed a previous inflammatory process.

 The diagnosis of TINU syndrome requires the presence of both AIN and uveitis, without other known systemic diseases that can cause either interstitial nephritis or uveitis [2]. Clinical findings include abnormal renal function (with renal insufficiency and proximal or distal tubular defects), abnormal urinalysis (with sterile pyuria, hematuria, proteinuria, aminoaciduria, normoglycemic glycosuria, phosphaturia, and acidification defects), and symptoms of a systemic illness lasting longer than 2 weeks. Laboratory findings may include eosinophilia, normochromic normocytic anemia, slightly abnormal liver function tests, and elevated ESR. TINU syndrome has been associated with the presence of ANA, ANCA, an autoantibody directed against renal tubular cells, rheumatoid factor, and hypocomplementemia. Elevations have been found in serum levels of beta-2 microglobulin and Krebs Von Den Lunge-6 (KL-6), a glycoprotein found in lung tissue of normal individuals as well as in the distal renal tubules of patients with TINU syndrome [20]. Ocular presentation of TINU is very uniform, with bilateral sudden-onset anterior uveitis, that is often asymptomatic. Uveitis may occur two months before, concurrently, and up to fourteen months after the onset of interstitial nephritis. Since the uveitis is predominantly anterior, main clinical findings include anterior chamber cells and flare, conjunctival injection, keratic precipitates, and dry eyes [21]. Posterior uveitis can also be seen, resulting in vitreous humour cells, chorioretinitis, intraretinal hemorrhages, retinal vascular sheathing, cotton wool spots, dilated retinal vessels, arteriovenous nicking, and retinal oedema [2]. Renal biopsy is a fundamental step to unequivocally establish the diagnosis of TINU syndrome. On light microscopy, typical findings include tubulointerstitial oedema and infiltration of inflammatory cells composed mainly of lymphocytes, plasma cells, and histiocytes. Eosinophils, neutrophils, and noncaseating granulomas are frequently seen. Glomerular and vascular structures are generally preserved. Findings with immunofluorescence and electron microscopy are also nonspecific [22]. 

 Although renal disease in patients with TINU is usually self-limited, we decided to use oral corticosteroids after having obtained bioptic evidence of tubulointerstitial nephritis. Renal function rapidly improved, and prednisone was progressively tapered without recurrence of either nephritis or uveitis. Patients with progressive renal insufficiency are typically treated with prednisone for three to six months [23]. Topical and systemic corticosteroids have been used for uveitis with success. However, recurrences and relapses of uveitis are common; infrequently, steroid-sparing immunosuppressive agents, such as cyclosporine, methotrexate, and mycophenolate mofetil, are needed. Patients must be carefully monitored, especially for development of diffuse vitreous opacities, which can decrease visual acuity [24]. 

 TINU syndrome is probably an underdiagnosed disorder, responsible for many cases of idiopathic anterior uveitis in young patients. The present experience shows that subclinical signs of uveitis must be actively searched especially in those patients who have tubulointerstitial disease of unknown origin.

## Figures and Tables

**Table 1:** Causes of acute tubulointerstitial nephritis.

Drugs (>75%)	
(i) Antibiotics: ampicillin, cephalosporins, ciprofloxacin, cloxacillin, methicillin, penicillin, rifampicin, sulfonamides, vancomycin.	
(ii) NSAIDs.	
(iii) Other: allopurinol, acyclovir, famotidine, furosemide, omeprazole, phenytoin.	
Infections (5%–10%)	
(i) Bacteria: Brucella, Campylobacter, Escherichia coli, Legionella, Salmonella, Streptococcus, Staphylococcus, Yersinia.	
(ii) Viruses: cytomegalovirus, Epstein-Barr virus, hantavirus, human immunodeficiency virus, polyomavirus, herpes simplex virus, hepatitis C virus.	
(iii) Other: Leptospira, Mycobacterium tuberculosis, Mycoplasma, Rickettsia, Schistosoma, Toxoplasma.	
Idiopathic (5%–10%)	
(i) Antitubular basement membrane antibodies.	
(ii) TINU.	
Associated with systemic diseases (10%–15%)	
(i) Sarcoidosis, Sjögren, systemic lupus erythematosus, Wegener's granulomatosis, rheumatoid arthritis, hypoparathyroidism, hyperthyroidism, lymphoproliferative disorders.	
